# Chloric Ether and Hydrochloric Ether

**Published:** 1855-07

**Authors:** John Aldridge


### ARTICLE IX.

*Chloric Ether and Hydrochloric Ether.* By JOHN ALDRIDGE M. D.

THERE is a preparation which has been known to, and employed by, the medical profession for many years under the names of "Chloric Ether," and "Terchloride of Carbon," either externally, for relieving pain in cancer and other diseases, or internally, as a diffusible stimulus. The names by which this liquid has been called are calculated to mislead as to its nature, it being nothing more or less, than a spirituous solution of chlo-

*Carpenter's General Physiology.

roform, made by mixing one part, by bulk, of chloroform, with seven parts of spirits of wine. This composition is well known to pharmaciens. It was mentioned in Dr. Austin's excellent "Retrospect of Pharmacy," published in the November number of the *Dublin Medical Quarterly* for 1847; and it is again stated in the 9th volume of the *Pharmaceutical Journal*, page 248. It was discovered by Mr. Guthrie, an American chemist, in 1831, and his process (as given in *Silliman's Journal* for 1832, and quoted by Pereira at page 1977 of the last edition of his great work) shows that it was a similar product that he obtained; for that process is calculated to afford *unwashed* chloroform, from which the spirit is not removed. From an erroneous conception of its composition, he named it "Chloric Ether," as from a subsequent mistake by Liebig it was called, "Terchloride of Carbon," but the name that it really deserves is that of "Spirits of Chloroform," it being, as I have stated, a solution of chloroform in spirits of wine.

But whatever confusion and difficulty may have been heretofore experienced in consequence of the erroneous names by which this medicine has been designated, that confusion and difficulty is now considerably increased by the proposal to substitute a totally different compound for it—a compound whose properties are very little known, and the use of which in medicine has been long since discarded. It is proposed to substitute for chloric ether (or spirits of chloroform, its proper name) a compound long known under the names of muriatic or hydrochloric ether. Now, it does not seem to be very valid reasoning to say, that because a substance is called by a wrong name, you should reject it, and use something which has been correctly denominated in its stead. If the medicine hitherto called "Chloric Ether" has been found valuable, you may quarrel with the name, but you retain the thing, and only change the appellation. But the proposition to which I refer suggests, in effect, that as chloric ether is an incorrect name for spirits of chloroform, you should in place thereof employ "Hydrochloric Ether."

This hydrochloric ether is very little known; but Pereira says it is a diuretic like sweet spirits of nitre. So far it does not appear to be a very good substitute for chloric ether. A preparation of it was contained in the Edinburgh Pharmacopœia for 1735, and was subsequently omitted. This does not look as if much confidence was placed in it.

As in some conversations with medical men in this city, I have found much obscurity and misunderstanding to exist no this subject, I believe the explanation which I have here given may be of service.—*Dub. Med. Press.*

